# The prevalence of antibodies against the HLA-DRB3 protein in kidney transplantation and the correlation with HLA expression

**DOI:** 10.1371/journal.pone.0203381

**Published:** 2018-09-07

**Authors:** Thomas H. P. M. Habets, Bouke G. Hepkema, Niels Kouprie, Melanie C. A. Schnijderberg, Tim C. van Smaalen, Laura B. Bungener, Maarten H. L. Christiaans, Gerard M. J. Bos, Joris Vanderlocht

**Affiliations:** 1 Department of Transplantation Immunology, Tissue Typing Laboratory, Maastricht University Medical Center +, Maastricht, The Netherlands; 2 Department of Internal Medicine, Division of Hematology, Maastricht University Medical Center +, Maastricht, The Netherlands; 3 Transplantation Immunology, Department of Laboratory Medicine, University of Groningen, University Medical Center Groningen, Groningen, The Netherlands; 4 Department of Surgery, Maastricht University Medical Center +, Maastricht, The Netherlands; 5 Department of Internal Medicine, Division of Nephrology, Maastricht University Medical Center +, Maastricht, The Netherlands; 6 CiMaas BV, Maastricht, The Netherlands; 7 Central Diagnostic Laboratory, Maastricht University Medical Center +, Maastricht, The Netherlands; Istituto di Ricovero e Cura a Carattere Scientifico Centro di Riferimento Oncologico della Basilicata, ITALY

## Abstract

Human leukocyte antigen (*HLA*)-*DRB3* is a functional HLA class II gene, which has a limited allele diversity in the human population. Furthermore, the *HLA-DRB3* gene is only present in a subset of individuals. Therefore, in organ transplantation, this HLA molecule is frequently mismatched between patient and graft donor and thus antibodies against this mismatched HLA molecule can develop. In this study, we aimed to evaluate the prevalence and reactivity of these antibodies and aimed to identify factors that underlie antibody formation against HLA-DRB3. We showed in our patient cohort that HLA-DRB3 antibodies are identified in about 7% of all patients that were screened with solid phase assays. In these assays, we observed multiple antibody reactivity patterns indicating that HLA-DRB3 harbours multiple epitopes. In those cases, where we succeeded at tracing back the induction of these antibodies to the molecular HLA typing of the immunogenic event, we noticed a different frequency of *HLA-DRB1* allele groups in the donors as compared to a control group. To a certain extent this distribution (*e*.*g*. HLA-DRB1*11 individuals) could be linked to an altered expression level. However, it also appears that different *HLA-DRB3* alleles (*e*.*g*. HLA-DRB3*01 group) vary in their immunogenicity without having an expression difference. In conclusion, our study provides information on the immunogenicity and reactivity patterns of antibodies against HLA-DRB3 in kidney transplantation, and it points towards the possibility of HLA expression as a factor underlying antibody formation.

## Introduction

Human leukocyte antigen (HLA) class II molecules, such as HLA-DR, play an important role in the presentation of processed peptides from extracellular pathogens to the T cell receptor (TCR) of CD4+ helper T cells [1, 2]. They are expressed on the surface of antigen presenting cells (APC) like B cells, macrophages, and dendritic cells [3, 4]. The HLA-DR molecules are heterodimers that consist of an alpha (α)-chain (encoded by *HLA-DRA*) that shows limited diversity and a beta (β)-chain (encoded by *e*.*g*. *HLA-DRB1*) that is highly polymorphic in the population [3, 5]. This high diversity influences the peptide presentation and as such determines an individuals’ ability to respond to a wide variety of pathogens. In addition, the allelic variation in the population is thought to be a result of natural selection and represents a mechanism by which the population is protected against rapidly evolving pathogens [6].

The *HLA-DRB1* gene was duplicated in evolution and subsequently some individuals have a second *HLA-DRB* gene that encodes a functional protein on a single haplotype [7, 8]. These associated HLA-DRB proteins are tightly associated with *HLA-DRB1* and are encoded by *HLA-DRB3*, *HLA-DRB4*, and *HLA-DRB5*. Which of the associated *HLA-DRB* genes is present on a single haplotype depends on which *HLA-DRB1* allele is present, although exceptions are described. The HLA-DRB1*03, *11, *12, *13, *14 groups of alleles have a *HLA-DRB3* gene, whereas the HLA-DRB1*04, *07, *09 groups of alleles have a *HLA-DRB4* gene, the HLA-DRB1*15, *16 alleles have a *HLA-DRB5* gene, and the HLA-DRB1*01, *08, *10, groups of alleles have no functional associated *HLA-DRB* gene [9, 10]. The associated *HLA-DRB* gene products form together with the conserved α-chain (encoded by *HLA-DRA*), associated HLA-DRB proteins. These associated *HLA-DRB* proteins are not present in all individuals, but only in a subset (*e*.*g*. *HLA-DRB3* is present in 43% of the caucasoid population). Furthermore, these associated *HLA-DRB* genes show allelic variation in the population, *e*.*g*. the *HLA-DRB3* gene shows modest allelic diversity with 145 alleles as compared to 2103 alleles of *HLA-DRB1* [10–14].

For HLA-DRB3 it has been shown that it contributes to antigen presentation and host defence. In addition, the allelic variation of HLA-DRB3 has been proven to influence peptide presentation. The most convincing evidence of this involves the presentation of the human platelet antigen 1a (HPA-1a). The presentation of this HPA-1a peptide is highly restricted to HLA-DRB3*01:01 [15]. Therefore, only carriers of this *HLA-DRB3* allele are at risk to develop HPA-1a antibodies, which can induce neonatal alloimmune thrombocytopenia (NAIT) and fetal-maternal alloimmune thrombocytopenia (FMAIT) [16–19]. Besides NAIT and FMAIT in which the presence of a specific *HLA-DRB3* allele is a prerequisite for disease susceptibility, HLA-DRB3 also contributes to the overall disease susceptibility for numerous autoimmune diseases. These diseases include myasthenia gravis, Graves’ disease, Crohns’ sarcoidosis, and primary sclerosing cholangitis [20–23].

In organ transplantation, it is well established that matching patients and donors with respect to the HLA molecules has a major impact on the transplant survival [24]. The HLA loci that have the most impact on transplant outcome are *HLA-DRB1* followed by *HLA-B* [25]. It is speculated that the importance of *HLA-DRB1* and *HLA-B* is related to their higher expression on the cell surface. There are studies that link the expression levels of different HLA molecules to an altered capacity to induce an immune response against viral pathogens. For example, the expression level of HLA-C affects the risk of graft versus host disease (GVHD) after hematopoietic stem cell transplantation (HSCT), but also the clinical outcome of human immunodeficiency virus infection and Crohn’s disease [26, 27]. Furthermore, high expression of HLA-DPB1 and mismatches in HLA-DRB3 after HSCT was associated with the risk of GVHD [28, 29].

Since the number of HLA mismatches is correlated with inferior transplant outcome, it is possible that the impact of HLA-DR matching is larger because *HLA-DRB1* is associated with the co-expressed *HLA-DRB* genes such as *HLA-DRB3*. Another factor contributing to the survival of transplanted organs is the occurrence of rejection episodes. Even though major improvements in the immunosuppressive strategies were made, rejection episodes are not preventive. At present, antibody mediated rejection (AMR) still plays a fundamental role in graft loss in a subset of kidney recipients. In AMR, antibodies directed against mismatched HLA molecules of the donor graft are associated with inferior transplant outcome [30–33]. Until now, there is limited insight in the prevalence and importance of antibody formation against HLA-DRB3 in solid organ transplantation (*e*.*g*. kidney). In addition, it remains unclear whether factors such as expression levels of mismatched HLA molecules of the donor induce antibody formation in transplant recipients.

In our study, we aimed to examine how frequent antibodies are observed against the HLA-DRB3 protein in a kidney transplant cohort. We analysed the antibody reactivity patterns and correlated the patterns to the immunizing event in order to establish whether certain *HLA-DRB3* alleles are more immunogenic. Furthermore, we determined the gene and protein expression of HLA-DRB3 and evaluated the graft outcome in recipients that develop HLA-DRB3 antibodies after transplantation.

## Materials and methods

### Sera from patients and ethics

We made use of routine antibody screening of patients who undergo or await organ transplantation in the Dutch transplant centers of Maastricht and Groningen. The collection, storage, and usage of tissue and patient data have been performed in agreement with FEDERA (Federation of Dutch University Medical Centers) Code of Conduct (federa.org); According to Dutch law, Institutional Review Board (IRB) approval was not required for scientific analysis of anonymous data. Additionally, the research described in this article is according to Dutch standards called ‘niet WMO plichtigheid’: medical scientific research that does not involve patients’ actions or behaviours. All patients who visited both hospitals are informed about the procedure that material can be used for scientific research without further consent if the research is according to this standard. Plus, the sera used in this study are defined as left-over from clinical purposes and further use for medical research purposes is in accordance to Dutch ethical regulations. **Maastricht:** A total of 1800 kidney patients were transplanted in the period 1982 to 2014. In 645 of these patients, sera were tested for the presence of HLA class II antibodies using solid phase assays. **Groningen:** We applied a comprehensive database search for patients (organ waiting list and after transplantation) in whom HLA-DRB3 specific antibodies were demonstrated.

### Control groups

To study which *HLA-DRB3* allele is the most immunogenic, we determined the *HLA-DRB3* high resolution typing of the immunizing events vs. a control group. Selection process of the control group: A total of 140 *HLA-DRB3* positive patients without HLA-DRB3 antibodies were anonymously and randomly selected using our Maastricht transplant center database.

To examine whether certain donors are more prone to induce an HLA-DRB3 antibody response, we determined the *HLA-DRB3*-linked *HLA-DRB1* distribution (HLA-DRB1*03, *11, *12, *13, and *14) in the donor group vs. a control group. Selection process of the control group: A total of 258 *HLA-DRB3* positive patients (without HLA-DRB3 antibodies) with an available *HLA-DRB1* typing were anonymously and randomly selected using our Maastricht transplant center database. Both control groups were separately selected, so there is no overlap in between these groups.

### Detection of HLA-DRB3 antibodies using the Luminex Single Antigen (LSA) assay

The HLA-DRB3 antibodies in the serum of patients were detected using the LABScreen SA HLA class II assay (One Lambda, Thermo Fisher, Canoga Park, CA, USA) and Lifecodes LSA class II assay (Immucor, Norcross, GA, USA) according to the manufacturers’ protocol. The LABScreen (Lot: 009) and Lifecodes (Lot: 01145C) LSA kits contained three microbeads coated with single HLA-DRB3 alleles that represent the three major allele groups of the HLA-DRB3 protein: HLA-DRB3*01:01, HLA-DRB3*02:02, and HLA-DRB3*03:01. In short, the microbeads coated with purified HLA class II molecules were incubated with patient serum for 30min. Subsequently, antibodies that bound to HLA-DRB3 proteins coated on the microbeads were detected with PE (phyco-erythrine)-conjugated goat anti-human IgG after an incubation of 30min at room temperature. After washing, all microbeads were measured with the Luminex 100 multiplex analyser (Luminex, Austin, TX, USA) and analysed using HLA Fusion v.3.4 (One Lambda) and MatchIT v.1.2 (Immucor) software. In S1a Fig, an illustration displays the LSA assay and the position of the HLA-DRB3 microbeads of both vendors. The microbeads with a MFI value ≥1000 were considered positive for HLA-DRB3.

### CDC assay to determine the panel reactive antibody

1 μL serum of the patient was incubated with a screening panel of 60 different lymphocyte-suspensions (1 μL suspension; 4 *10^6^ cells/mL) for 30min (RT). Subsequently, 5 μL rabbit complement (Life Technologies) was added and incubated for 60min (RT) to initiate lymphocyte lysis via CDC. The addition of 5 μL FluoroQuench (One Lambda) for 10min (RT) allowed discrimination of intact versus lysed cells by means of automated fluorescence microscopy (Leica). In this way, the panel-reactive antibody (PRA) can be determined. In case we were not able to determine the PRA, we calculated the virtual PRA (vPRA) using the Eurotransplant reference laboratory vPRA calculator. This tool calculates the vPRA based on unacceptable antigens for HLA-A, -B, -C, -DR, and -DQ. The vPRA database contains data of 6870 donors with a complete HLA typing.

### Sequence based typing (SBT) of the *HLA-DRB3* gene

The sequence based typing of *HLA-DRB3* was previously described [34]. In short, the amplification reactions of *HLA-DRB3* were performed in a total mix volume of 30 μL that included 67 mM Tris-HCl (pH 8.8) (Merck, Darmstadt, Germany), 5% glycerol (Alfa Aesar, Karlsruhe, Germany), 1.5 mM MgCl_2_ (Life Technologies), 0.01% Tween 20 (Merck), 16.6 mM ammonium sulphate (Merck), 0.2 mM of each dNTP (GE Healthcare, Diegem, Belgium), 15 pmol of each primer (Sigma-Aldrich, Zwijndrecht, The Netherlands), 0.1 μg/μL cresol red (Sigma-Aldrich), 300 ng DNA, and 1.4 U expand high fidelity enzyme mix (Roche, Basel, Switzerland). The cycling conditions consisted of 2min at 94°C; 10 cycles of 15s at 94°C, 30s at 63°C, 4min at 68°C; followed by 10 cycles of 15s at 94°C, 30s at 60°C, 6min at 68°C; followed by 10 cycles of 15s at 94°C, 30s at 60°C, 10min at 68°C, and 7min at 68°C. The amplified products were visualized and checked for size on a 1.5% agarose gel (Life Technologies) with a concentration of 0.5 μg/mL ethidium bromide (Sigma-Aldrich).

The amplified products were sequenced using 4 sequence primers that covered exon 2 and exon 3 of *HLA-DRB3*. The total sequence volume of 10 μL included 6 μL water, 0.5 μL primer (5 pmol), 1 μL BigDye Terminator v1.1 mix, 1.5 μL BigDye Terminator sequencing buffer (Life Technologies), and 1 μL purified amplification product. The cycling conditions consisted of 1min at 96°C; 25 cycles of 10s at 96°C; 5s at 50°C, and 4min at 60°C. After sequencing, the products were purified with Sephadex G-50 (GE) and electrophoresed on the ABI 3730 DNA analyzer (Applied Biosystems, Foster City, CA, USA). The sequence data were analysed using Seqpilot v.3.5.2 software (JSI, Kippenheim, Germany).

### B cell isolation for *HLA-DRB1* and *HLA-DRB3* quantitative PCR

During routine diagnostics, splenocytes from deceased donors were isolated for crossmatch and typing purposes. The ethical regulations are described in the section ‘Sera from patients and ethics’. The anonymous human material used in this study are defined as left-over and the use of this material for research purposes is in accordance to Dutch ethical regulations. In short, splenic parts from deceased donors were homogenized into a single cell suspension using a gentleMACS dissociator (Miltenyi Biotec, Leiden, The Netherlands) (S1 Table). The mononuclear cells were separated from the single cell suspension using Lymphoprep. The obtained cells were frozen in RPMI-1640 medium containing 10% DMSO, 10% FCS and 1% PenStrep and stored in liquid nitrogen. Upon thawing, 10 *10^6^ splenocytes were washed and resuspended. Subsequently, B cells were isolated using the CD19 positive B cell isolation kit per manufacturers’ protocol (Miltenyi Biotec). The obtained B cells were stained for purity after the isolation using CD19 (clone HIB19, BD, APC), CD3 (clone UCHT1, BD, Horizon V450), CD56 (clone B159, BD, PE-Cy7) and CD14 (clone M5E2, BD, FITC) and measured using flow cytometry (BD FACS Canto II). The purity of the B cells exceeded ≥99% (S3 Fig; *n* = 5).

### Quantitative PCR to determine the relative mRNA expression of *HLA-DRB1* and *HLA-DRB3*

Total RNA was isolated from lysed B cells (positive B cell isolation kit) using the RNeasy Mini kit according to manufacturers’ protocol (Qiagen). Residual genomic DNA was removed by DNase I treatment followed by reverse transcription using random hexonucleotide primers and Superscript III Reverse Transcriptase according to a standard protocol (Invitrogen). The forward and reverse primers for quantitative PCR are shown in S2 Table. To prevent DNA amplification, both the *HLA-DRB1* and *HLA-DRB3* primers were designed to span exon-exon boundaries with a maximal amplification length of 300bp. A standard curve of a reference sample (cDNA of a total B cell fraction) was generated for relative quantification. The real-time (RT)PCR was performed with SYBR green detection (SensiMix SYBR, Bioline Reagents, London, UK) using the iCycler iQ (Biorad Laboratories, Hercules, CA, USA) and 10 pmol of the specific primer. The PCR program consisted of 10min initial heating at 95°C, followed by 35 cycles of amplification (30s at 95°C, 20s at 62°C, 20s at 72°C) and a heating up to 92°C to create a melting curve (increased 0.5°C/7s). The data represent the expression of the gene of interest normalized to *HuPo* (human acidic ribosomal protein) or *GAPDH* [35], which were used as reference genes in our study (S2 Table).

### Assessment of the cell surface expression of HLA-DRB3 using flow cytometry

Splenocytes from deceased donors were used to determine the cell surface expression of the HLA-DRB3 protein (S1 Table). Spleens were homogenised to a single cell suspension, separated with Lymphoprep, and the cells were stored in liquid nitrogen. At the moment of flow cytometric assessment, 10 *10^6^ splenocytes were thawed, washed, resuspended, and counted. The flow cytometric procedure is shown in S5 Fig. In short, a total of 1 *10^5^ splenocytes were blocked with heat-inactivated fetal calf serum (FCS) (20min, RT). Subsequently, mouse monoclonal antibody 7.3.19.1 specific for epitope 77N (Thermo Fischer) that is only present on HLA-DRB3 (with the exception of HLA-DRB1*03) proteins was incubated for 30min. As control condition the IgG_2b_ isotype control was added (clone 27–35, BD) to check for non-specific binding. The B cell fraction was further specified with CD19 (clone HIB19, BD, APC). The monoclonal 7.3.19.1 antibody was detected with a PE labelled goat anti-mouse Ig antibody (20min, polyclonal, BD). In between all incubation steps the cells were washed with buffer (PBS 1X, 1% FCS, 0.02% NaN_3_). Prior to staining of mouse anti-human CD19, mouse serum (20min, Dako) was added to block the binding sites of the goat anti-mouse Ig antibody. A life to death marker 7-AAD was added before measurement (BD). The cells were measured and analysed using BD FACSCanto II and FACSDiva software. To standardize for the fluorescence intensity irrespective of the instrument and software, PE labelled beads were used (Bangs Laboratories Inc., Fishers, IN, USA). The mean fluorescent intensities (MFI) were adjusted to molecules of equivalent soluble fluorochromes (MESF) units [36].

### Figures and statistics

The statistical analyses were performed in GraphPad Prism Pro v.6.01 (GraphPad Software, La Jolla, CA, USA). The figures were made in Graphpad and combined in Adobe Illustrator (Adobe Systems, San Jose, CA, USA). The difference in allele frequencies of HLA-DRB1 was determined using the Chi-squared test. The Mann-Whitney t-test was used to define a statistical significance between groups. The Kaplan-Meier survival curves were compared using the log-rank (Mantel-Cox) test. For all analyses, a P value <0.05 (*) was statistically significant. P-values <0.01 were graphically presented as (**).

## Results

### The prevalence and Luminex Single Antigen reactivity patterns of HLA-DRB3 antibodies in the sera of organ transplant recipients

**Fig 1:**
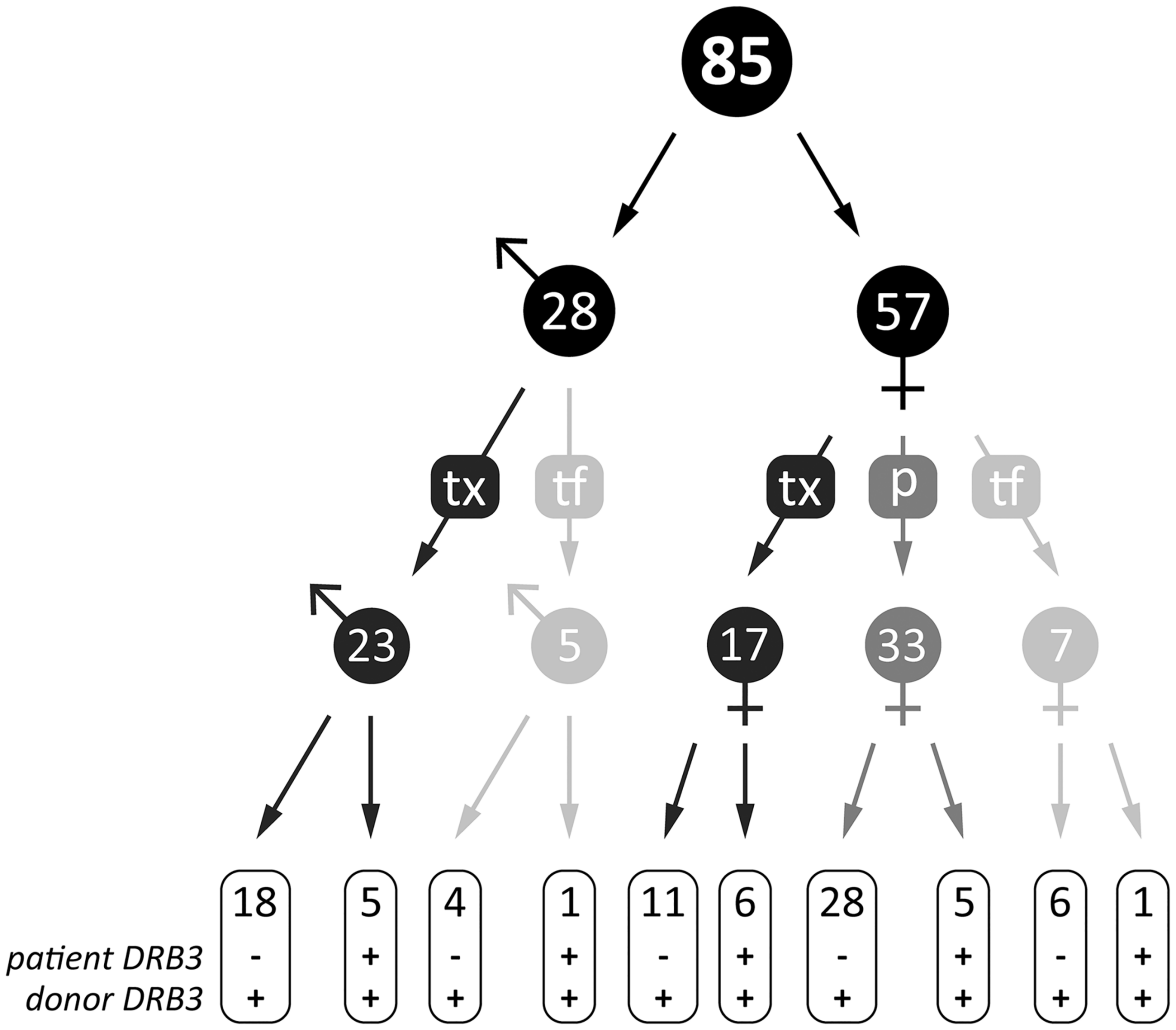
Characterization of 85 positive patients with HLA-DRB3 antibodies based on their immunizing event and whether patients and donors (children in case of pregnancy) were *HLA-DRB3* gene carrier or not. We made use of routine antibody screening of patients that undergo or await organ transplantation in the Dutch transplant centers of Maastricht and Groningen. We detected in a total of 85 patients (n = 28 male and n = 57 female; black symbol) HLA-DRB3 antibodies after different immunizing events such as kidney transplantation (tx; dark grey), transfusion (tf; light grey), or pregnancy (p; medium grey). We also determined whether the patients and donors (children in case of pregnancy) were *HLA-DRB3* gene carrier or not. Mismatched cases are shown as—for *patient DRB3* and + for *donor HLA-DRB3*; and matched cases are shown as + for *patient DRB3* and + for *donor HLA-DRB3*.

To study the prevalence of antibodies against HLA-DRB3, we made use of routine antibody screening of patients that undergo or await kidney transplantation in the transplant centers of Maastricht and Groningen. In Maastricht, 1800 kidney patients were included and in 645 of these patients, sera were tested in solid phase assays (LSA: Luminex Single Antigen) in addition to cytotoxicity testing. A retrospective LSA analysis of these 645 patients showed that 43 kidney patients (7%) were positive for HLA-DRB3 antibodies. In addition, in the center of Groningen we applied a search in the local transplant database for patients in which HLA-DRB3 were detected in routine antibody screening. Using this approach, we identified 42 additional patients with HLA-DRB3 antibodies. Notably, 6 out of these 42 patients were waiting for their first lung or heart transplantation. Since transplantation was not the immunogenic event inducing HLA-DRB3 antibody formation, we consider these 6 patients as patients on the waiting list for organ transplantation in whom pregnancy and/or transfusion is the event that triggered HLA-DRB3 antibody formation. All cases where we identified organ transplantation as immunogenic event were kidney transplant recipients. As shown in Fig 1, we characterized a total of 85 positive patients based on their immunizing event (transplantation, transfusion, and/or pregnancy) and whether patients were carrying the *HLA-DRB3* gene or not.

Presence of allele-specific HLA class II antibodies in the sera of kidney patients was assessed by use of LSA microbead assays (Maastricht from One Lambda and Groningen from Immucor). As shown in S1a Fig, the LSA kits from One Lambda and Immucor contained three microbeads coated with HLA-DRB3*01:01, HLA-DRB3*02:02, and HLA-DRB3*03:01 proteins. Although these LSA assays are similar in terms of methodology, both centers use a different LSA vendor. Therefore, the LSA kits may have different antibody reactivity as a result of the purification and coating of HLA-DRB3 proteins on the microbeads. We tested to what extent the LSA kits from both vendors were consistent in terms of antibody reactivity patterns. The data of this technical validation are presented in the supporting information (S1 File and S1b Fig). The conclusion of this technical validation was that both kits are reproducible in terms of determining whether a serum contains antibodies against HLA-DRB3 or not. Though, both kits displayed discrepancies when detecting antibodies against specific HLA-DRB3 alleles, especially against HLA-DRB3*03:01. We observed this in the sera of HLA-DRB3 carriers.

**Fig 2:**
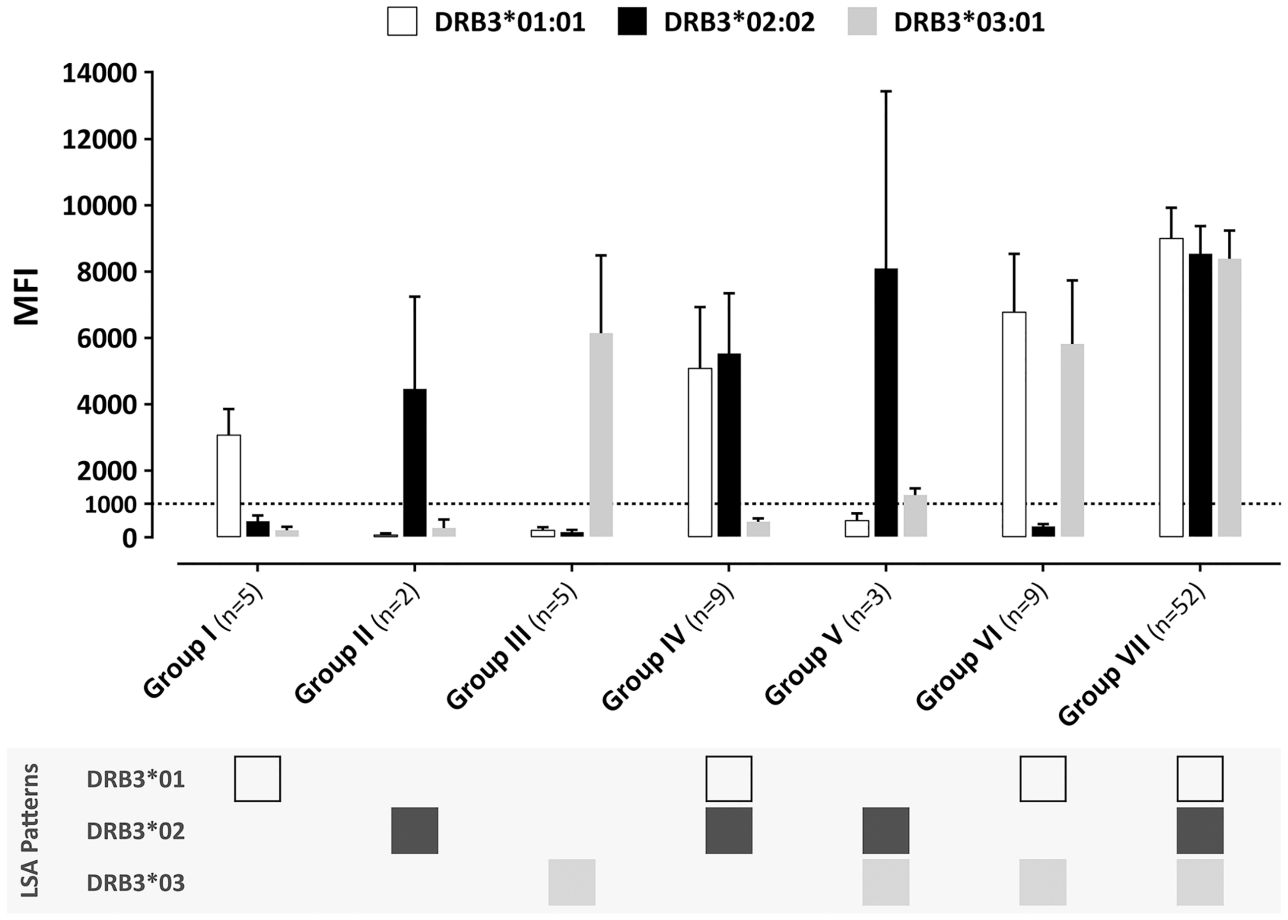
The reactivity patterns of HLA-DRB3 antibodies against Luminex SA microbeads. The reactivity patterns of HLA-DRB3 antibodies against three LSA microbeads (One Lambda and Immucor) in 85 patients. The groups represent all possible binding combinations of HLA-DRB3 antibodies in the sera of patients to one, two, or three microbeads coated with HLA-DRB3*01:01, HLA-DRB3*02:02, and HLA-DRB3*03:01 proteins. A MFI value of ≥1000 was considered to be positive for HLA-DRB3. The data is plotted as mean and SEM.

As shown in Fig 2, examination of the LSA reactivity patterns for 85 positive patients showed all seven HLA-DRB3 antibody reactivity patterns (group I to VII), confirming that the HLA-DRB3 protein has multiple epitopes. Most of the patients (52 out of 85) displayed an antibody reactivity to all three HLA-DRB3 microbeads (group VII) (mean fluorescent intensity (MFI) value ±8500). Furthermore, 67 out of 85 patients who showed antibodies against HLA-DRB3 are individuals who do not carry the *HLA-DRB3* gene, while 18 patients were *HLA-DRB3* gene carriers. In terms of antibody reactivity patterns, these two groups act different: the sera of patients who do not carry the *HLA-DRB3* gene (52 out of 67) showed an antibody reactivity primarily against all three microbeads (S2a Fig) (group VII), while the sera of patients who carry the *HLA-DRB3* gene showed reactivity with specific DRB3 allele groups (S2b Fig).

### Immunization with HLA-DRB3*01 and HLA-DRB3*02 displayed more induction of HLA-DRB3 antibodies in kidney patients as compared to HLA-DRB3*03

**Fig 3:**
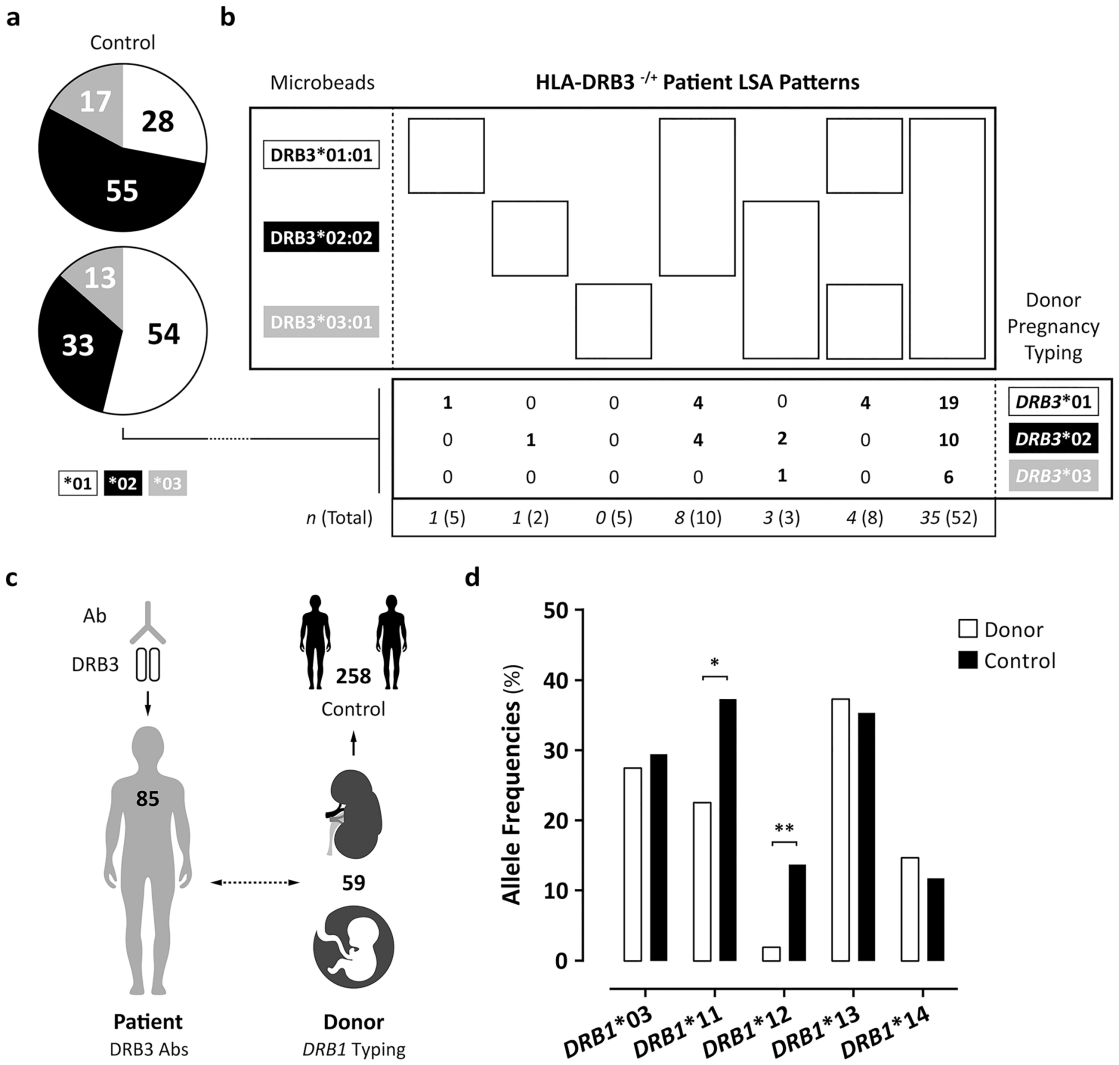
The immunologic events of HLA-DRB3*01, *02, *03 and the allele frequencies of *HLA-DRB1* in the donors. a, b. The immunologic events defined as the *HLA-DRB3* (*01, *02, *03) typing of the graft donors or children (pregnancies) and the reactivity patterns of HLA-DRB3 antibodies against LSA microbeads HLA-DRB3*01:01, *02:02, and *03:01 that were detected in HLA-DRB3 negative and positive kidney patients (*n* = 52). The percentages of the patients’ LSA reactivity pattern that correspond with the *HLA-DRB3* donor typing are shown in the pie chart. The distribution of the *HLA-DRB3* alleles of the graft donors or children are compared with a control group (*HLA-DRB3* positive individuals without HLA-DRB3 antibodies). c. In 85 kidney patients HLA-DRB3 antibodies were detected. With respect to the donor group, 59 graft donors or children were typed for *HLA-DRB1*. These *HLA-DRB1* alleles (linked to *HLA-DRB3*) were compared with a control group in a case-control setting. d. The allele frequencies of HLA-DRB1*03, *11, *12, *13, and *14 are displayed as percentage. The difference between donor and control groups was calculated using the Chi-squared test. The P values of <0.05 (*) and <0.01 (**) were statistically significant.

To examine which *HLA-DRB3* allele is the most immunogenic in terms of antibody induction, we determined the *HLA-DRB3* typing of the immunizing events (graft donor or child in case of pregnancy). We did an HLA typing of the graft donors or children for HLA-DRB3*01, HLA-DRB3*02, and HLA-DRB3*03 using SBT (exon 2 and 3). In 52 out of 85 patients we succeeded to identify the immunizing event and was it possible to obtain DNA for molecular typing of *HLA-DRB3*, while we had no DNA of the other 33 patients. In 28 cases the immunizing event was a transplantation (graft donor), and in 24 cases a pregnancy. In Fig 3a, we showed that 54% of the microbead reactivity patterns in the patients had an immunizing event with HLA-DRB3*01, whereas 33% had HLA-DRB3*02, and 13% had HLA-DRB3*03. The distribution of HLA-DRB3*01 and HLA-DRB3*02 was significantly different compared to the control group with 140 *HLA-DRB3* positive individuals without HLA-DRB3 antibodies. In this control group, we observed that 28% had HLA-DRB3*01, 55% had HLA-DRB3*02, and 17% had HLA-DRB3*03. In Fig 3b, the correlation between the microbead reactivity pattern and the nature of the immunizing event is shown. We did not notice reactivity patterns that did not include the microbead coated with the sensitizing HLA-DRB3 molecule. The reactivity pattern that recognized all three microbeads was observed most frequent (35 out of 52). Furthermore, we observed that all possible reactivity patterns containing the microbead coated with the *HLA-DRB3* allele of the sensitizing event were observed.

Taken together, our data suggest that in kidney patients with HLA-DRB3 antibodies, HLA-DRB3*01 was more immunogenic than HLA-DRB3*02 and HLA-DRB3*03. In addition, the correlation of the donor typing with the microbead reactivity pattern showed that allele group specific reactivity is a consequence of immunization and not an artefact of solid phase assays. Therefore, also the allelic diversity of HLA-DRB3 is immunogenic.

### The frequencies (measured by the HLA-DRB1 alleles that are associated with HLA-DRB3) are different in the donor group as compared to a control group

To address the hypothesis whether certain donors present peptides differently and are more prone to induce an HLA-DRB3 antibody response, we investigated the *HLA-DRB1* distribution in the donor group and a control group. The expression of HLA molecules can be dependent on the haplotype organization. In case of broad antigens *HLA-DRB3*, *HLA-DRB4*, and *HLA-DRB5* the expression levels can be studied by analysing the linked *HLA-DRB1* gene. We assessed whether the allele frequencies of the *HLA-DRB1* alleles (HLA-DRB1*03, *11, *12, *13, and *14) that are linked with *HLA-DRB3* of the immunizing event (donors or children in case of pregnancy) showed a discrepancy in distribution as compared to control individuals. To this end, we compared the distribution of HLA-DRB1*03, *11, *12, *13, and *14 alleles in the immunizing event group to a total of 258 kidney transplant recipients carrying the *HLA-DRB3* gene without the presence of HLA-DRB3 antibodies (control). As illustrated in Fig 3c, in 59 out of the 85 patients who had HLA-DRB3 antibodies we had DNA to perform *HLA-DRB1* typing. As shown in Fig 3d, we observed a reduced allele frequency of HLA-DRB1*11 and HLA-DRB1*12 in the donor group as compared to the control group. In addition, the allele frequencies of HLA-DRB1*03, HLA-DRB1*13, and HLA-DRB1*14 were similar in the donor group as compared to the control group. The altered distribution of the *HLA-DRB1* alleles may indicate that certain *HLA-DRB1* alleles are more or less prone to induce antibodies in patients with HLA-DRB3 antibodies.

### The relative mRNA and protein expression of HLA-DRB1 and HLA-DRB3

**Fig 4:**
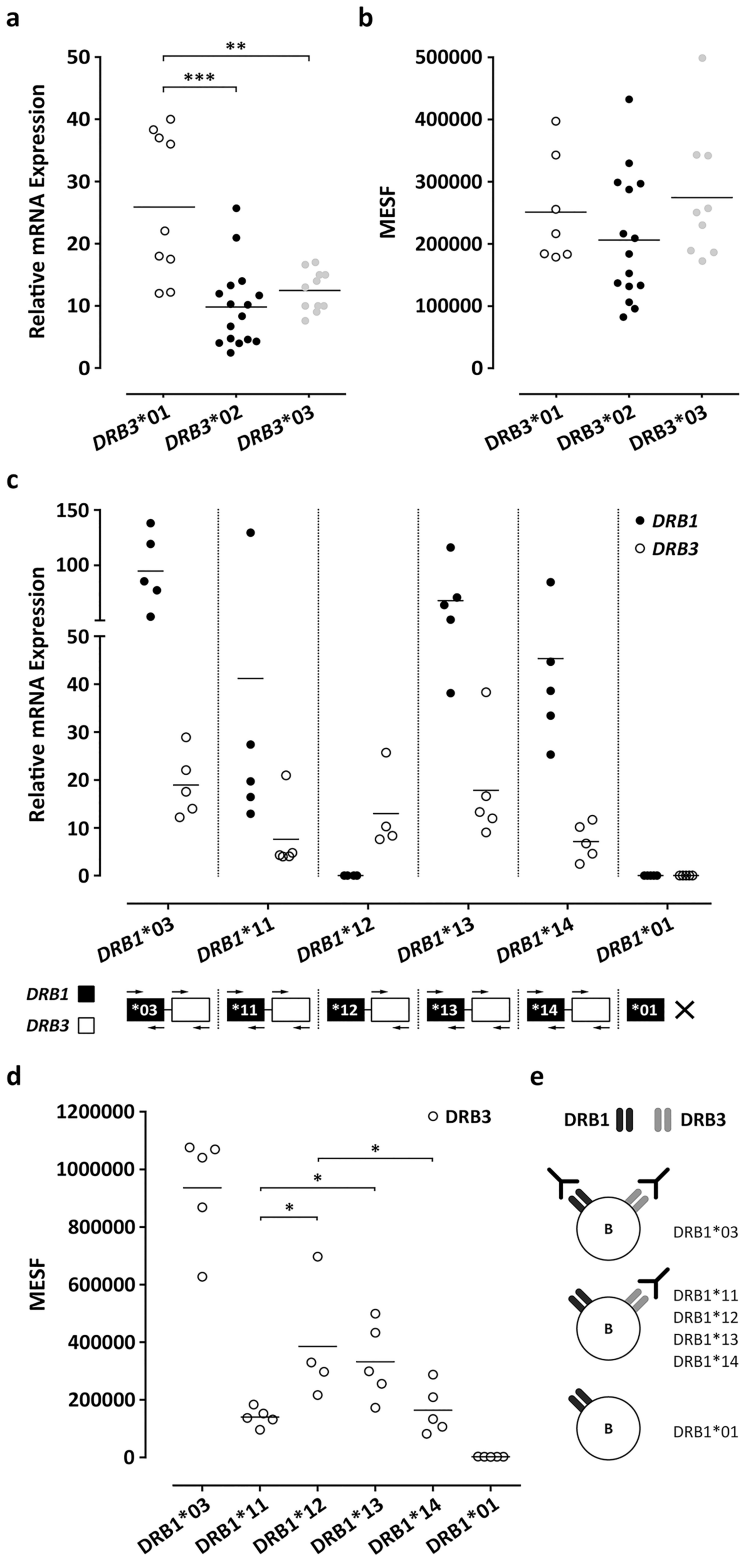
The relative mRNA and protein expression of HLA-DRB1 and HLA-DRB3. a. The relative mRNA expression of HLA-DRB3*01 (*n* = 9), *02 (*n* = 16), and *03 (*n* = 11) in isolated B cells from deceased donors. The relative mRNA expression was determined by quantitative PCR (Q-PCR) and the values were normalized to reference gene *HuPo*. b. The cell surface expression of HLA-DRB3*01 (*n* = 7), *02 (*n* = 15), and *03 (*n* = 9) on B cells. Splenocytes from the donors were incubated with monoclonal 7.3.19.1 antibody, which binds to the specific epitope 77N that is present on HLA-DRB3*01, *02, and *03 (and HLA-DRB1*03). The B cells were gated using a fluorescently labelled CD19 antibody and the MFI values were corrected to MESF units using PE labelled beads. c. The relative mRNA expression of HLA-DRB1*03, *11, *13, and *14 and *HLA-DRB3* in isolated B cells from deceased donors (*n* = 5 per group) determined by Q-PCR (normalized to *HuPo*). HLA-DRB1*01 was used as negative control for HLA-DRB1/3 primers. A primer for HLA-DRB1*12 was not included, therefore the products are not amplified. d, e. The expression of HLA-DRB3 on B cells with HLA-DRB1*03, *11, *12, *13, and *14 (*n* = 5 per group). HLA-DRB1*01 was used as negative control. The cell surface expression is shown as MESF units. The *HLA-DRB1* typing is shown in S1 Table. Mann-Whitney t-test. The P values of <0.05 (*), <0.01 (**), and <0.001 (***) were significant.

To determine whether expression variances contribute to antibody induction, we assessed the relative mRNA and protein expression of HLA-DRB1 and HLA-DRB3. The relative mRNA expression of the *HLA-DRB1* and *HLA-DRB3* genes was determined with quantitative PCR. We developed specific forward primers for *HLA-DRB1* alleles HLA-DRB1*03, HLA-DRB1*11, HLA-DRB1*13, and HLA-DRB1*14 and for *HLA-DRB3* (S2 Table). HLA-DRB1*01 (no secondary *HLA-DRB* gene) was used as negative control for both primers (illustration Fig 4c). The *HLA-DRB1* primer did not allow amplification of HLA-DRB1*12. Since the allele frequency of HLA-DRB1*12 is very low in the caucasoid population, we did not develop separate primers. The relative mRNA expression of HLA-DRB1*03, *11, *13, and *14 and *HLA-DRB3* was assessed in B cells, and the values were normalized to the reference gene *HuPo*.

As shown in Fig 4a, B cells from individuals with HLA-DRB3*01 displayed a higher relative mRNA expression than HLA-DRB3*02 (P<0.001) and HLA-DRB3*03 (P<0.01). Notably, in 4 individuals with HLA-DRB3*01 we observed a higher relative mRNA expression than the other individuals. There was no difference between HLA-DRB3*02 and HLA-DRB3*03. With regard to *HLA-DRB1*, we observed that the relative mRNA expression of all *HLA-DRB1* alleles (HLA-DRB1*03, *11, *13, *14) was higher as compared to *HLA-DRB3* (Fig 4c). The expression difference of *HLA-DRB1* and *HLA-DRB3* had an average of 5.3 ±1.9 fold. However, we did not notice a substantial difference in relative mRNA expression among the different *HLA-DRB1* alleles (HLA-DRB1*03, *11, *13, *14) and their linked *HLA-DRB3*. Comparable results were observed when using *GAPDH* as reference gene (5.3 ±1.9 fold data; S4 Fig).

Subsequently, we determined whether the HLA-DRB3 protein expression is different across these *HLA-DRB1* alleles. We measured the HLA-DRB3 expression (monoclonal 7.3.19.1 antibody) on the cell surface of B cells using flow cytometry (S5a Fig). We preferred to use a monoclonal antibody, since HLA molecules are highly polymorphic and due to this feature polyclonal antibodies or sera may show increased cross-reactivity with other HLA molecules. This makes it difficult to assess the cell surface expression of a specific HLA molecule. The B cells were selected with CD19 positivity (S5b Fig). We included 5 donors with HLA-DRB1*03 as positive control, since the monoclonal antibody will bind to HLA-DRB1 and HLA-DRB3. In addition, we selected 5 donors with HLA-DRB1*01 as negative control, since the monoclonal antibody will not bind to HLA-DRB1*01, and HLA-DRB3 is absent (HLA-DRB1*01 is not linked to *HLA-DRB3*). This approach allows us to compare the HLA-DRB3 expression in donors with HLA-DRB1*11, *12, *13, and *14.

As shown in Fig 4b, there was no difference in cell surface expression of *HLA-DRB3* alleles HLA-DRB3*01, HLA-DRB3*02, and HLA-DRB3*03. This indicates that the increased relative mRNA expression of HLA-DRB3*01 did not result in elevated cell surface expression. With respect to the *HLA-DRB1* alleles, we observed a reduced expression of the HLA-DRB3 protein in 5 donors with HLA-DRB1*11 as compared to HLA-DRB1*12, and HLA-DRB1*13, but not to HLA-DRB1*14 (Fig 4d). In case of HLA-DRB1*14, the expression of the HLA-DRB3 protein is lower as compared to HLA-DRB1*12, but not to HLA-DRB1*11 and HLA-DRB1*13. In addition, we showed that the monoclonal 7.3.19.1 antibody reacted with HLA-DRB1 and HLA-DRB3 in 5 donors with HLA-DRB1*03 and that 5 donors with HLA-DRB1*01 showed no antibody reactivity. The antibody reactivity is illustrated in Fig 4e.

Taken together, HLA-DRB3*01 showed a higher relative mRNA expression than HLA-DRB3*02 and HLA-DRB3*03. Though, there was no difference in protein expression among the *HLA-DRB3* alleles. Likewise, we did not observe substantial differences in relative mRNA expression of *HLA-DRB3* between the linked *HLA-DRB1* alleles. Nevertheless, we observed a reduced protein expression of HLA-DRB3 in case of HLA-DRB1*11 and *14.

### The graft survival in transplant recipients with or without HLA-DRB3 antibodies

**Fig 5:**
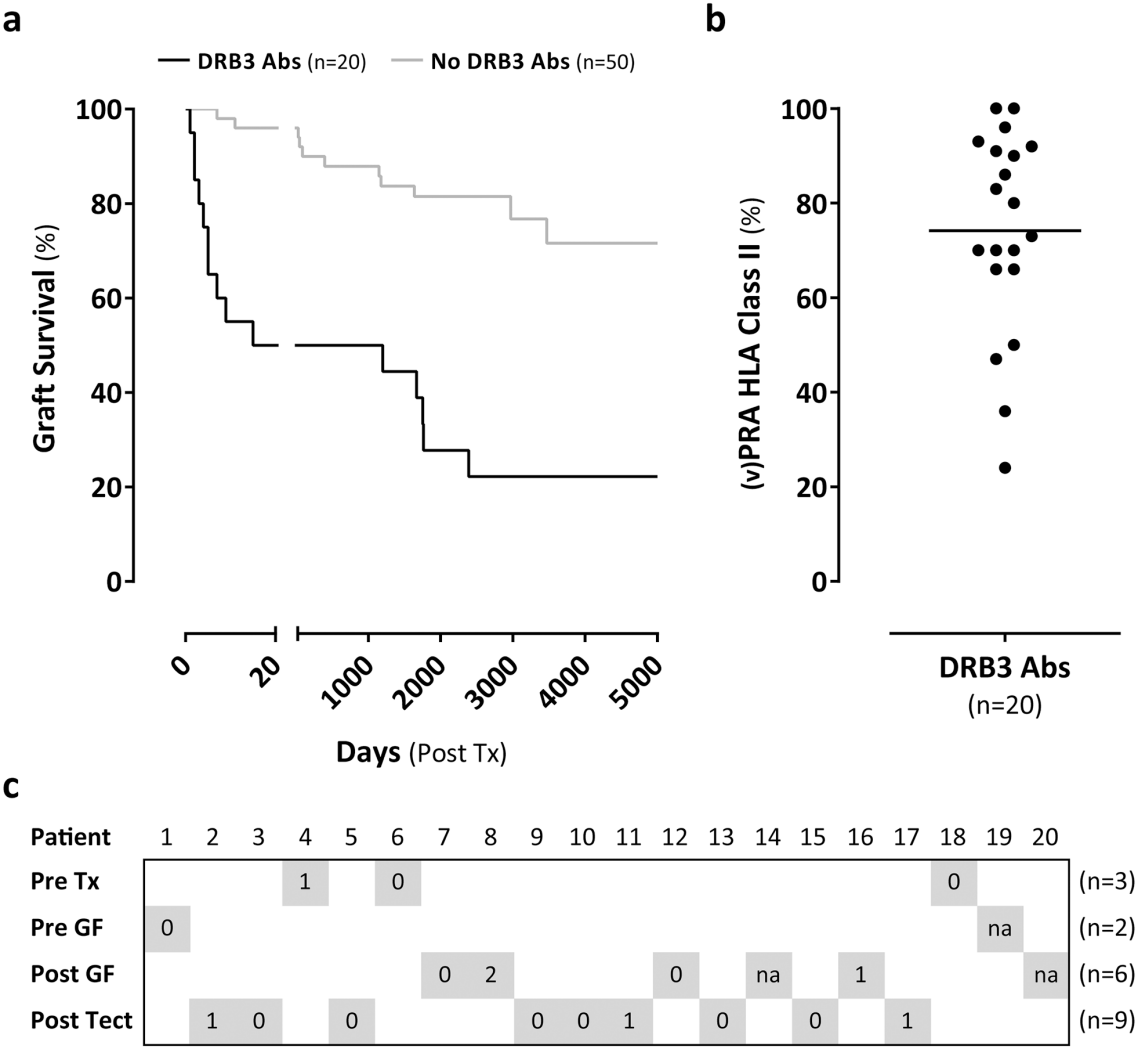
Graft survival in transplant recipients with or without HLA-DRB3 antibodies. a. Kaplan-Meier survival curve of transplanted grafts in recipients with (*n* = 20) or without (*n* = 50) HLA-DRB3 antibodies. The loss of a transplanted graft (survival) was defined as the date of graft failure after transplantation. Log-rank (Mantel-Cox) test with P<0.0001. b. The (v)PRA HLA class II percentage of the recipients with HLA-DRB3 antibodies. The (v)PRA was determined in the same serum sample as the HLA-DRB3 antibodies. (v)PRA = (virtual) panel reactive antibodies. c. Overview panel indicating the moment when HLA-DRB3 antibodies were found in the 20 recipients with HLA-DRB3 antibodies. The light grey squares show whether the antibodies were found before transplantation (pre tx), before graft failure (pre GF), after graft failure (post GF), or after transplantectomy (post tect). Other detected donor specific antibodies are shown as a number or as not available (na) in the squares.

In order to assess whether HLA-DRB3 antibodies develop in a specific subgroup of patients, we retrospectively determined graft outcome in patients (*n* = 20) who displayed antibodies after kidney transplantation. In 17 out of 20 patients the HLA-DRB3 antibodies developed after transplantation. As shown in Fig 5a, 10 patients that developed HLA-DRB3 antibodies demonstrated a transplant failure (return to dialysis) within 20 days after transplantation. Notably, patients who showed these antibodies after transplantation displayed at the same time antibodies against many other HLA molecules, which is indicated by a panel-reactive antibody (PRA) of 74 ±21% (Fig 5b). The determination of the PRA using CDC assays is clinically relevant as it indicates the presence of complement-fixing antibodies against 74% of the Caucasoid donor population. Importantly, as shown in Fig 5c, in 15 out of 20 patients the HLA-DRB3 antibody was detected after graft failure and transplantectomy. In 3 patients we observed preformed HLA-DRB3 antibodies prior to transplantation and in 2 patients we observed these antibodies prior to graft loss but not prior to transplantation. Furthermore, in 15 out of 20 patients with HLA-DRB3 antibodies data was available of the donor origin of the transplanted grafts. All grafts were derived from post-mortem donors further defined as 5 grafts from donation after brain death (DBD) and 10 grafts from donation after cardiac death (DCD) donors.

## Discussion

HLA-DRB3 belongs to the HLA class II beta-chain paralogues and is present in a subgroup of the human population. Antibodies against HLA-DRB3 are frequently encountered in organ transplantation recipients. In case HLA-DRB3 would be taken along as unacceptable antigen during allocation, this will have a considerable impact on the donor frequency. Furthermore, antibodies that are observed in patients that do not react with all tested HLA-DRB3 variants in solid phase assays may have implications for HLA typing during allocation. We aimed to examine the occurrence and reactivity of antibodies in organ transplant recipients. To this end, we included a total of 85 transplant patients at two transplantation centres displaying HLA-DRB3 antibodies. In a comprehensive screening of 645 patients with solid phase assays we observed that 7% of patients develop antibodies throughout their time on the waiting list. We observed that HLA-DRB3 antibodies develop upon different kinds of immunogenic events including kidney transplantation, pregnancy, and transfusion. In 79% of patients with antibodies against HLA-DRB3 did not carry the *HLA-DRB3* gene, whereas 21% of patients that also show these antibodies are carriers of the *HLA-DRB3* gene. This indicates that not only the presence of the *HLA-DRB3* gene is immunogenic but also the allelic variation of the *HLA-DRB3* gene in different carriers.

In terms of antibody reactivity, we demonstrated in solid phase assays that multiple reactivity patterns are observed, reflecting that HLA-DRB3 harbours multiple epitopes. The reactivity patterns observed in patients that do not carry *HLA-DRB3* are different from those observed in *HLA-DRB3* carriers. The pattern where all microbeads are recognized is the most abundant pattern among all patients, and this is only observed in patients that do not carry the *HLA-DRB3* gene. In *HLA-DRB3* carriers the HLA-DRB3 antibodies mainly reacted with one or two microbeads. This is a clear indication that the epitopes, which are preferentially recognized in *HLA-DRB3* carrying patients are different from those patients that do not carry the *HLA-DRB3* gene. A limitation of these LSA assays is that there are only three microbeads covering the major HLA-DRB3 alleles. This is insufficient to determine all epitopes that might be important for the immunogenicity of HLA-DRB3, and therefore specific microbeads may be developed that cover more HLA-DRB3 alleles.

When we correlated the microbead reactivity pattern with the high resolution *HLA-DRB3* typing of the immunizing events, we observed in almost all cases that the microbead carrying the antigen of immunization was included in the reactivity pattern. However, we did observe a vendor bias when comparing microbead-specific reactivity patterns with two different vendors. This is primarily confined to individuals carrying the *HLA-DRB3* gene. All microbeads showed discrepancies in reactivity, but most were observed with the microbead containing HLA-DRB3*03:01. The differences in reactivity can be a result of the purification and coating of HLA-DRB3 proteins on the surface of microbeads or this reactivity can also be affected by a different protein conformation of the coated HLA-DRB3 proteins [37]. Reed *et al*. performed an extensive analysis of solid phase multiplex bead assays in the field of HLA antibodies, in which they showed that the MFI was various according to the vendor and microbead type [38]. Currently, it remains unclear what the clinical relevance of these allele-specific antibodies is, nevertheless our results indicate that these antibodies develop as a result of immunization and that they are not artefacts in solid phase detection systems.

When assessing the graft outcome in kidney transplant recipients who develop HLA-DRB3 antibodies, we observed that these patients demonstrate an inferior graft outcome as compared with a control group that did not develop these HLA-DRB3 antibodies. It should be noted that this is not considered proof of the clinical relevance of these antibodies, since in the majority of patients (15 out of 20) these antibodies developed after graft failure or transplantectomy and are therefore not involved in graft loss. Because these HLA-DRB3 antibodies arise at the same time when a wide antibody response against a variety of HLA molecules, may indicate that HLA-DRB3 antibodies are induced preferentially when a robust antibody response is observed. A short graft survival followed by rapid transplantectomy is apparently associated with the occurrence of these antibodies. Notably, the HLA-DRB3 antibodies that are induced as a result of other immunizing events are not invariably associated with a diverse antibody response (data not shown). In addition, the inferior graft outcome in these recipients can be partly attributable to the donor origin, since all these patients received post-mortem grafts from either DBD or DCD donors. The quality of post-mortem grafts is associated with inferior transplant outcome as compared to living graft donors [39]. Although these data provide an indication about the role of HLA-DRB3 antibodies in kidney transplantation, this study was not intended to assess graft outcome as it shows limitations such as the number of included patients and that our population is homogeneously Caucasian. With respect to the clinical relevance, an additional analysis was performed by the Dutch PROCARE consortium [40]. Only 50 transplantations (out of 577) were HLA-DRB3 mismatched as compared to 85 without HLA-DRB3 mismatch. This analysis showed that an HLA-DRB3 mismatch was not associated with an inferior graft outcome (data not shown). Importantly, increasing the number of patients in an international multicenter study is essential to gain more insight in the overall prevalence of HLA-DRB3 antibodies and the clinical relevance of these antibodies in transplantation.

Since the induction of an antibody response may be dependent on the dose of the antigen on the kidney transplant, we questioned whether there are differences in the mRNA and protein expression of different donors depending on their *HLA-DRB3* typing or based on their *HLA-DRB1* allele. We made use of a depository of HLA-typed frozen splenocytes from which we isolated B cells to perform our expression studies. Importantly, the disadvantage of using this material may be that the total process of harvesting splenocytes and isolating B cells may influence the stability of the mRNA and proteins. Still, we observed comparable mRNA and protein levels of HLA-DRB3 when we used fresh blood samples (data not shown). Furthermore, our findings are in line with previous studies that reported that the relative mRNA expression of *HLA-DRB1* is always higher than *HLA-DRB3* [41, 42]. Emery *et al*. showed that this difference in expression between *HLA-DRB1* and *HLA-DRB3* is caused by the presence of specific motifs (to which transcription factors bind) in the X box region of the promotors of these genes [43]. Although *HLA-DRB*3 is always lower expressed than *HLA-DRB1*, we observed consistent differences in the HLA-DRB3 expression across different *HLA-DRB1* alleles. We did not address whether this expression difference is due to variances in the promotor of *HLA-DRB3* or whether regulatory motifs *in cis* outside the promotor region are involved. Though, we observed an altered distribution of *HLA-DRB1* alleles in the donors that lead to the induction of HLA-DRB3 antibodies as compared to an HLA-DRB3+ population without HLA-DRB3 antibodies. The reduced preponderance of HLA-DRB1*11 in the donors was in line with a reduced cell surface expression of HLA-DRB3 in case of HLA-DRB1*11. Although this expression study is limited in size, it provides support for the hypothesis that the capacity to induce an antibody response to the HLA-DRB3 protein of a mismatched HLA molecule may be dependent on the expression level of the molecule. These data add up to previous reports that HLA expression levels contribute to transplant outcome. Petersdorf *et al*. reported that the expression level of HLA-C and HLA-DP is of significant importance in influencing the strength of alloimmune responses in recipients that had mismatched HLA loci [26, 28]. Furthermore, Zilinska *et al*. showed that HLA-G mRNA expression was increased in recipients with acute rejection such as AMR and T cell-mediated rejection as compared to patients with dysfunctional non-rejected grafts [44].

In addition to our observation that the expression level of a mismatched HLA-DRB3 molecule may be involved in antibody formation we also observed that not all *HLA-DRB3* alleles are equally immunogenic. When comparing the distribution of the *HLA-DRB3* alleles in the immunizing events with a control population we observed a preponderance of the HLA-DRB3*01 group of alleles. This may point towards an increased immunogenicity of HLA-DRB3*01 as compared to HLA-DRB3*02 and HLA-DRB3*03. Furthermore, we observed that also the exact *HLA-DRB3* allele determines the capacity to induce an antibody response and it is not plausible that a difference in expression is the cause of this discrepancy. Faner *et al*. did not observe a difference in relative mRNA expression of HLA-DRB3*01 and *02 in both B cells and monocytes [10]. Although we observed that the relative mRNA expression of HLA-DRB3*01 in B cells was higher as compared to HLA-DRB3*02 and *03, this did not translate into a difference in the cell surface expression of HLA-DRB3.

In conclusion, our study showed that HLA-DRB3 antibodies are frequently detected in kidney patients after different immunologic events including transplantation, pregnancy, and transfusion. The induction of these antibodies after transplantation or transplant failure is associated with a wide allosensitization. We demonstrated that HLA-DRB3 and its’ allelic diversity is immunogenic, and that HLA-DRB3 harbours multiple epitopes. Importantly, we provide a first indication that the risk of developing an antibody response against HLA-DRB3 is to a certain extent dependent on HLA expression. However, our study awaits confirmation in an international multicenter study especially with regard to the clinical relevance of these antibodies, which remain unclear. Furthermore, it should be investigated whether the HLA-DRB3*01 group of alleles is more immunogenic than the other groups; and whether lower expression of HLA-DRB3 in HLA-DRB1*11 haplotypes may underlie the underrepresentation in the immunizing donors.

## Supporting information

S1 Fig The reactivity patterns of HLA-DRB3 antibodies against Luminex SA microbeads using two vendors. (TIF)

S2 Fig The reactivity patterns of HLA-DRB3 alloantibodies or intra-allele antibodies against LSA microbeads in 85 patients. (TIF)

S3 Fig The purity staining of isolated B cells from splenocytes using flow cytometry. (TIF)

S4 Fig The relative mRNA expression of *HLA-DRB1* and *HLA-DRB3* in B cells determined with quantitative PCR and normalized to *GAPDH*. (TIF)

S5 Fig Schematic overview of the flow cytometric procedure and gating strategy to determine B cells that express HLA-DRB3. (TIF)

S1 Table The *HLA-DRB1* typing of splenocytes from deceased donors. (PDF)

S2 Table The *HLA-DRB1* (*03, *11, *13, and *14), *HLA-DRB3* (*01, *02, and *03), *HuPo*, and *GAPDH* primers (forward and reverse) used for quantitative PCR. (PDF)

S1 File Supporting information. (PDF)
