# Supplementary figures and images for: The prevalence of antibodies against the HLA-DRB3 protein in kidney transplantation and the correlation with HLA expression
Source: PLoS One. 2018 Sep 7;13(9):e0203381. doi: 10.1371/journal.pone.0203381 (PMC6128541; doi:10.1371/journal.pone.0203381)

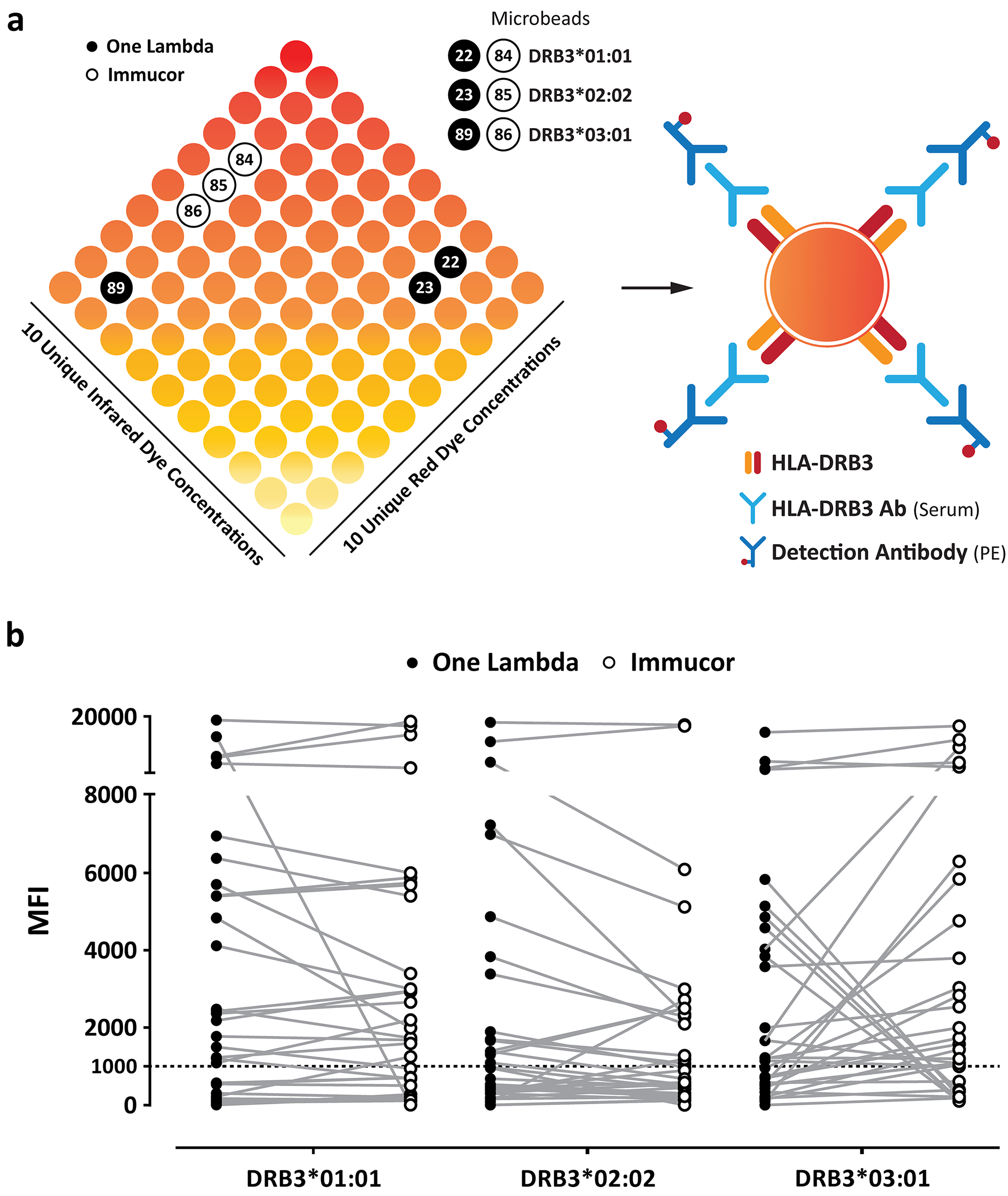

Supplement: S1 Fig — (TIF) [file pone.0203381.s001.tif]

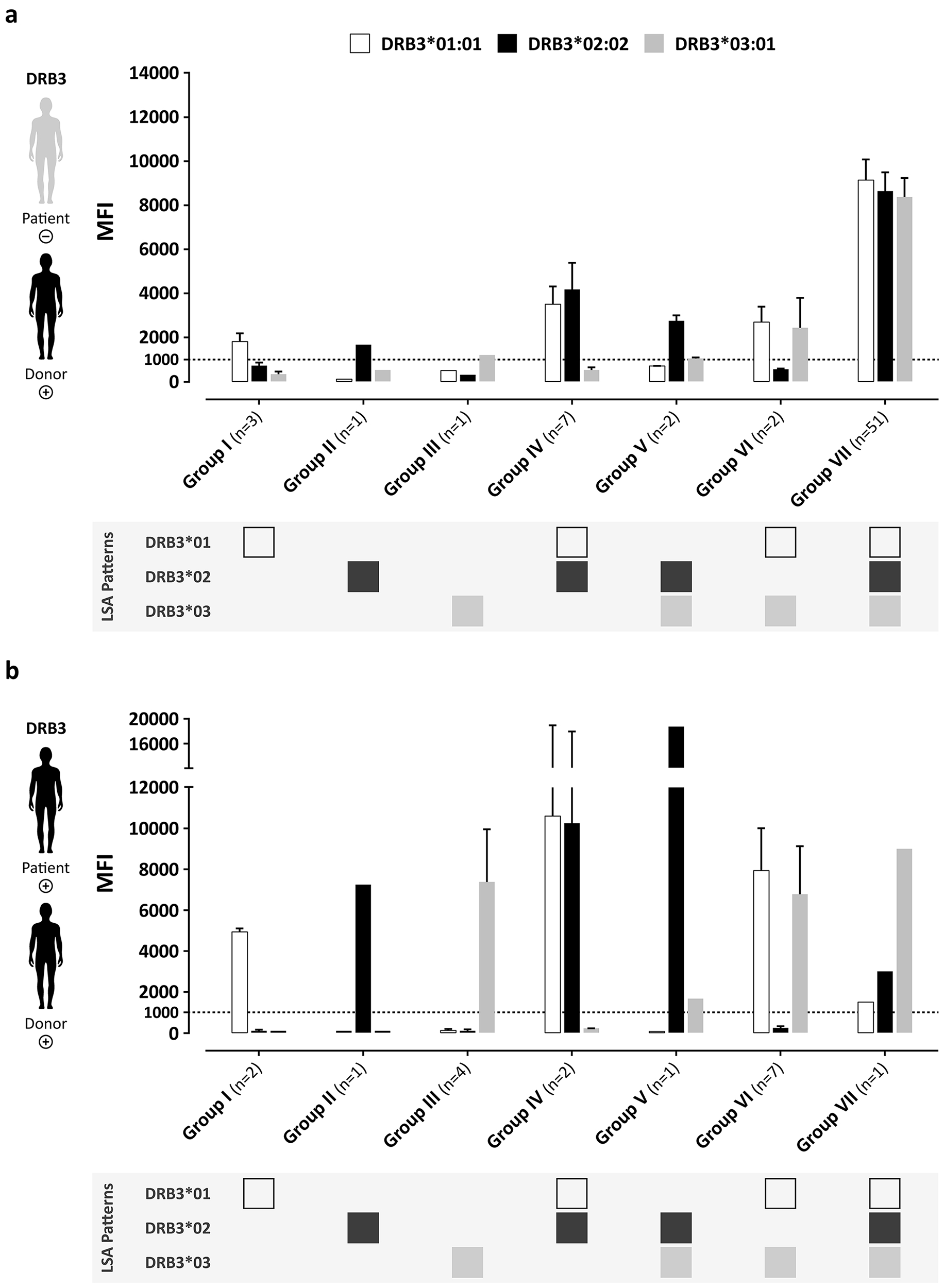

Supplement: S2 Fig — (TIF) [file pone.0203381.s002.tif]

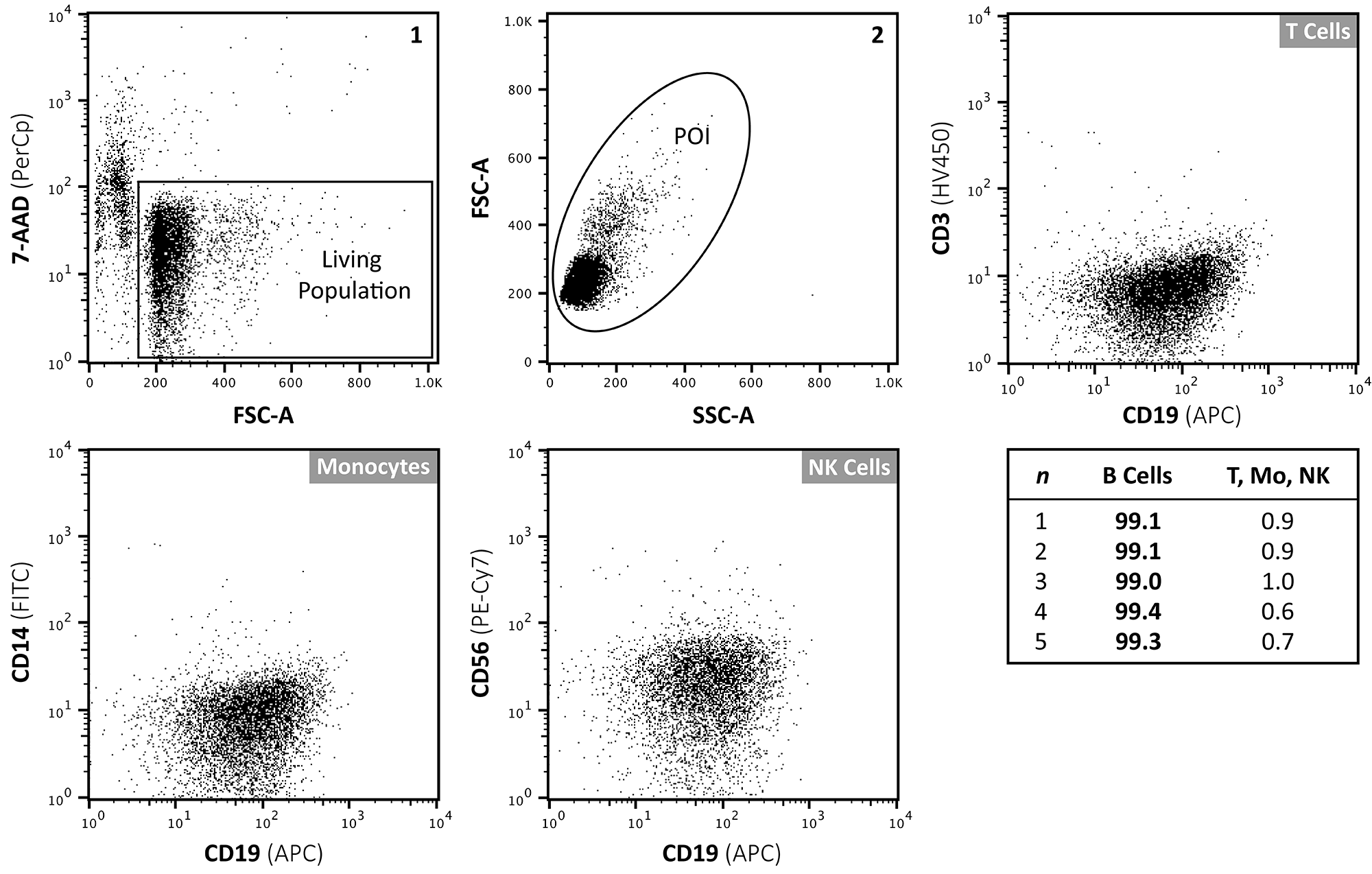

Supplement: S3 Fig — (TIF) [file pone.0203381.s003.tif]

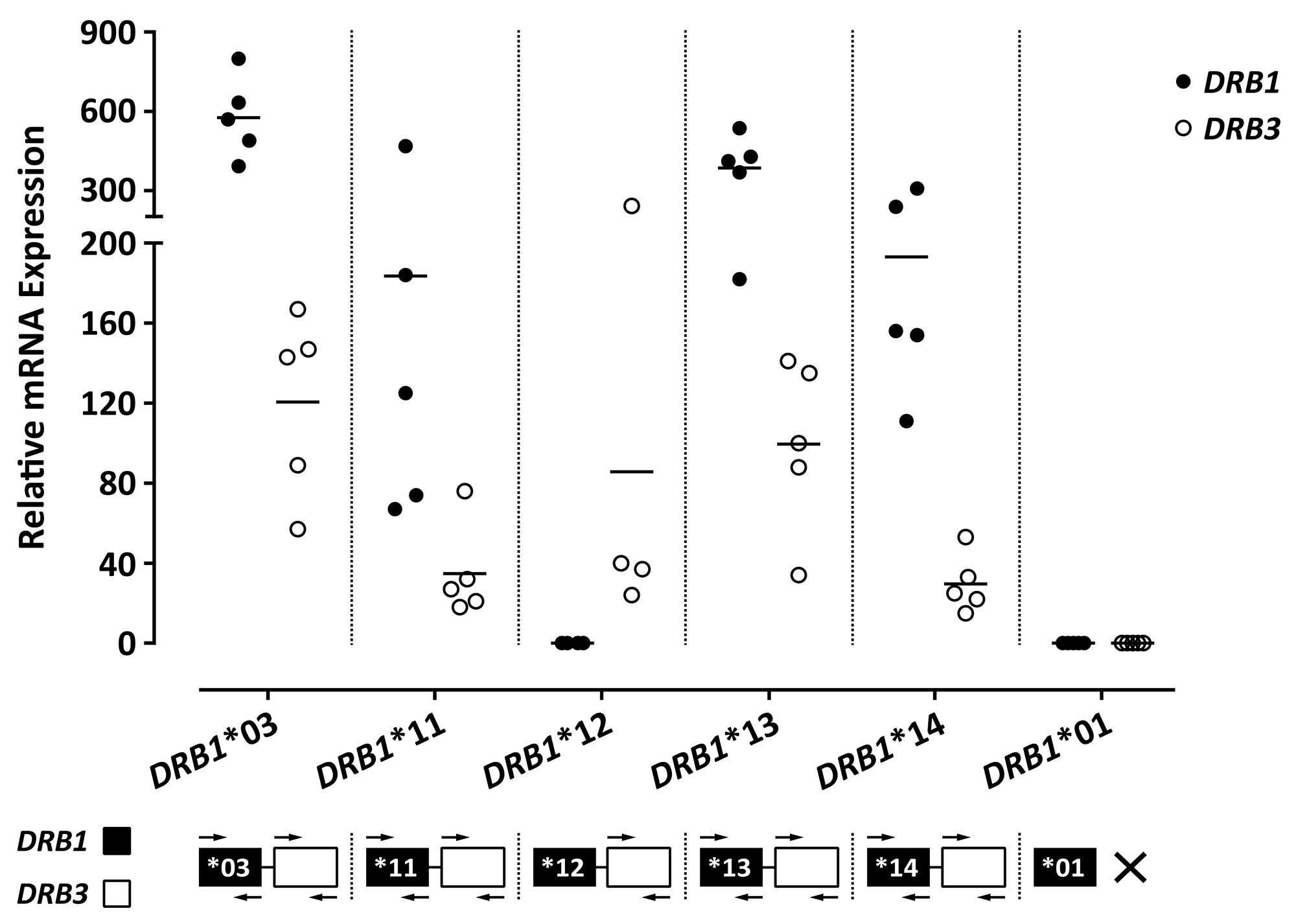

Supplement: S4 Fig — (TIF) [file pone.0203381.s004.tif]

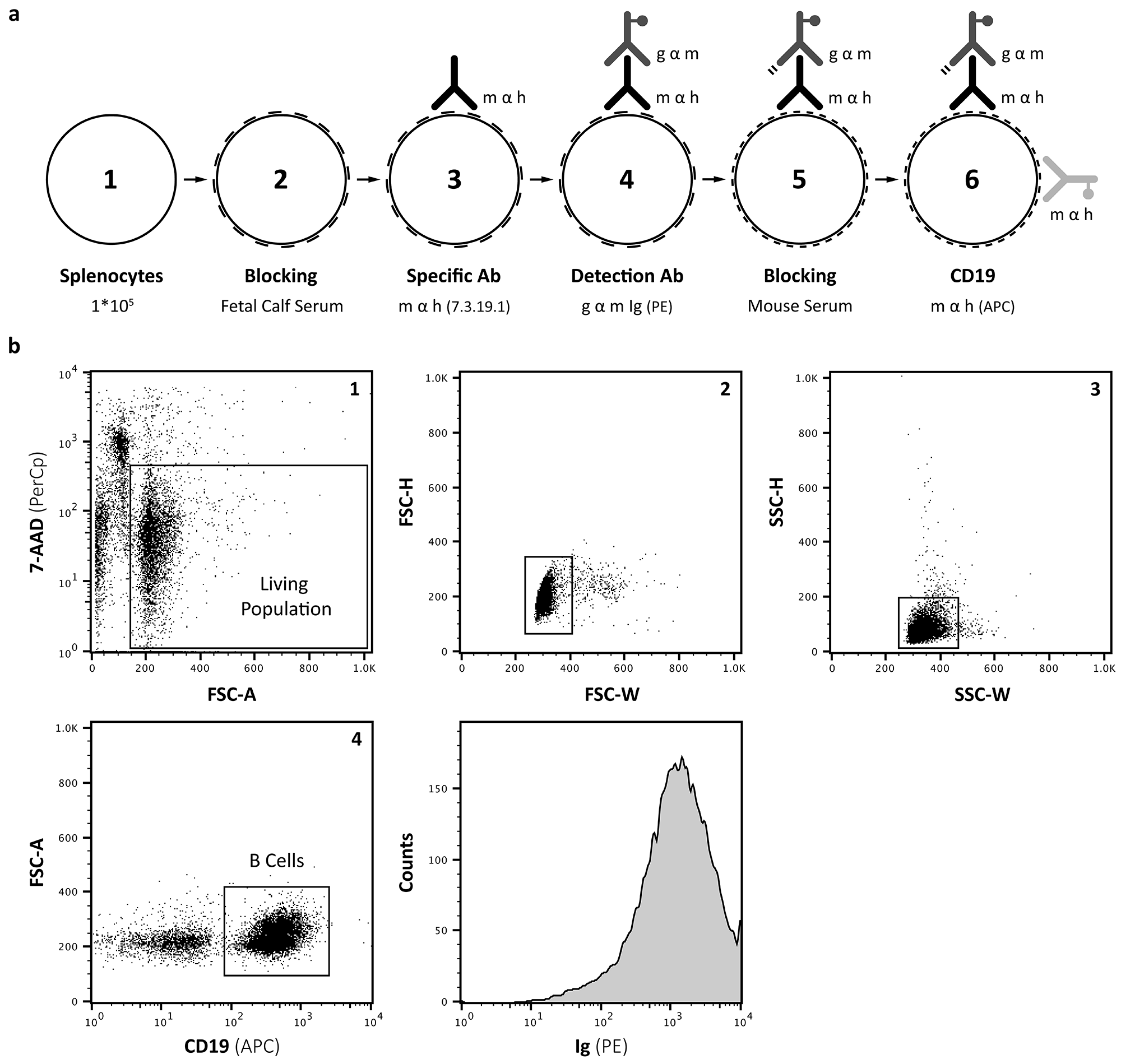

Supplement: S5 Fig — (TIF) [file pone.0203381.s005.tif]
