# Supplementary material for: The prevalence of antibodies against the HLA-DRB3 protein in kidney transplantation and the correlation with HLA expression
Source: PLoS One. 2018 Sep 7;13(9):e0203381. doi: 10.1371/journal.pone.0203381 (PMC6128541; doi:10.1371/journal.pone.0203381)
Supplement: S1 Table — (PDF) [file pone.0203381.s006.pdf]

# The prevalence of antibodies against the HLA-DRB3 protein in kidney transplantation and the correlation with HLA expression

**S1 Table. The *HLA-DRB1* typing of splenocytes from deceased donors.**

| DR (Ser) | *DRB1* (DNA) | Donor | Allele | Allele | He/Hom |
|---|---|---|---|---|---|
| **DR1** | ***DRB1**01** | 1 | *01 | | hom |
| | | 2 | *01 | *08 | he |
| | | 3 | *01 | | hom |
| | | 4 | *01 | *10 | he |
| | | 5 | *01 | | hom |
| **DR3** | ***DRB1**03** | 1 | *03 | | hom |
| | | 2 | *01 | *03 | he |
| | | 3 | *03 | | hom |
| | | 4 | *03 | | hom |
| | | 5 | *03 | | hom |
| **DR5** | ***DRB1**11** | 1 | *11 | *15 | he |
| | | 2 | *11 | | hom |
| | | 3 | *11 | *15 | he |
| | | 4 | *01 | *11 | he |
| | | 5 | *11 | *15 | he |
| | ***DRB1**12** | 1 | *01 | *12 | he |
| | | 2 | *01 | *12 | he |
| | | 3 | *12 | | hom |
| | | 4 | *12 | *15 | he |
| **DR6** | ***DRB1**13** | 1 | *13 | | hom |
| | | 2 | *13 | | hom |
| | | 3 | *13 | | hom |
| | | 4 | *13 | | hom |
| | | 5 | *13 | | hom |
| | ***DRB1**14** | 1 | *08 | *14 | he |
| | | 2 | *08 | *14 | he |
| | | 3 | *14 | *15 | he |
| | | 4 | *08 | *14 | he |
| | | 5 | *10 | *14 | he |

Ser = serology, He = heterozygous, Hom = homozygous
