# Supplementary material for: The prevalence of antibodies against the HLA-DRB3 protein in kidney transplantation and the correlation with HLA expression
Source: PLoS One. 2018 Sep 7;13(9):e0203381. doi: 10.1371/journal.pone.0203381 (PMC6128541; doi:10.1371/journal.pone.0203381)
Supplement: S2 Table — (PDF) [file pone.0203381.s007.pdf]

# The prevalence of antibodies against the HLA-DRB3 protein in kidney transplantation and the correlation with HLA expression

**S2 Table. The *HLA-DRB1* (*03, *11, *13, and *14), *HLA-DRB3* (*01, *02, and *03), *HuPo*, and *GAPDH* primers (forward and reverse) used for quantitative PCR.**

Forward 5' primers

| Gene | Alleles | 5' primer (5' - 3') | Location | Exon |
|---|---|---|---|---|
| *HLA-DRB1* | *03, *11, *13, *14 | GTTTCTTGGAGTACTCTACGTC [1] | 104-125 | Exon 2 |
| *HLA-DRB3* | *01, *02, *03 | GACCACGTTTCTYGGAGCT | 97-116 | Exon 2 |
| *HuPo* | n/a | ACGGGTACAAACGAGTCCTG | n/a | n/a |
| *GAPDH* | n/a | GCTCTCCAGAACATCATCCCTGCC | n/a | n/a |

Reverse 3' primer

| Gene | Alleles | 3' primer (5' - 3') | Location | Exon |
|---|---|---|---|---|
| *HLA-DRB1* | *03, *11, *13, *14 | CTGACTTCAATGCTGCCTGG | 457-476 | Exon 3 |
| *HLA-DRB3* | *01, *02, *03 | CTGACTTCAATGCTGCCTGG | 457-476 | Exon 3 |
| *HuPo* | n/a | TATCCTCGTCCGACTCCTCC | n/a | n/a |
| *GAPDH* | n/a | CGTTGTCATACCAGGAAATGAGCTT | n/a | n/a |

HLA = human leukocyte antigen, HuPo = human acidic ribosomal protein
Primer [1] is adapted from [45]. Location is indicated as position in the DNA sequence.

45. Voorter CE, Rozemuller EH, de Bruyn-Geraets D, van der Zwan AW, Tilanus MG, van den Berg-Loonen EM. Comparison of DRB sequence-based typing using different strategies. Tissue Antigens. 1997;49(5):471-6.
