# Supplementary material for: The prevalence of antibodies against the HLA-DRB3 protein in kidney transplantation and the correlation with HLA expression
Source: PLoS One. 2018 Sep 7;13(9):e0203381. doi: 10.1371/journal.pone.0203381 (PMC6128541; doi:10.1371/journal.pone.0203381)
Supplement: S1 File — (PDF) [file pone.0203381.s008.pdf]

The prevalence of antibodies against the HLA-DRB3 protein in kidney transplantation and the correlation with HLA expression

S1 File. Supporting information.

Validation of antibody reactivity patterns against three LSA HLA-DRB3 microbeads using two different vendors (One Lambda and Immucor).

Both centers use a different LSA vendor as described previously and as a consequence the LSA kits may have different antibody reactivity. Therefore, we examined to what extent the LSA kits were consistent in terms of antibody reactivity patterns. A pilot study of 10 serum samples with reactivity to one, two, or all three microbeads indicated that only the patients with antibody reactivity to one or two microbeads demonstrated discrepancies across the two vendors (data not shown). However, as shown in Fig S1b, we identified 32 sera that did not show reactivity with all three microbeads. In order to determine whether the antibody reactivity pattern was consistent across the vendors we tested these sera with both vendors. Although, we are aware of showing the mean fluorescent intensity (MFI) values which are dependent on the vendor, our intention was to detect false positive or false negative results. In these tests, 29 out of 32 samples showed reactivity against HLA-DRB3 with both vendors. In one of the samples the antibody reactivity was borderline with one vendor and just below the threshold with the other vendor. In the other two cases, we observed antibody reactivity with only one microbead that was completely negative with the other vendor. Among the 29 out of 32 samples that showed antibody reactivity against HLA-DRB3 with both vendors we identified 12 aberrant microbead reactivities. Interestingly, 10 out of 12 cases with aberrant reactivities were patients who were *HLA-DRB3* gene carriers. We observed discrepancies in all microbeads, however the most were observed in HLA-DRB3*03:01 coated microbeads.
